# Head-to-head comparisons of enhanced CT, ^68^Ga-PSMA-11 PET/CT and ^18^F-FDG PET/CT in identifying adverse pathology of clear-cell renal cell carcinoma: a prospective study

**DOI:** 10.1590/S1677-5538.IBJU.2023.0312

**Published:** 2024-02-07

**Authors:** Shao-Hao Chen, Bo-Han Lin, Shao-Ming Chen, Qian-Ren-Shun Qiu, Zhong-Tian Ruan, Ze-Jia Chen, Yong Wei, Qing-Shui Zheng, Xue-Yi Xue, Wei-Bing Miao, Ning Xu

**Affiliations:** 1 The First Affiliated Hospital of Fujian Medical University Department of Urology Urology Research Institute Fuzhou China Department of Urology, Urology Research Institute, The First Affiliated Hospital of Fujian Medical University, Fuzhou, China; 2 Fujian Medical University Department of Urology National Region Medical center Fuzhou China Department of Urology, National Region Medical center, Binhai Campus of the First Affiliated Hospital, Fujian Medical University, Fuzhou, China; 3 The First Affiliated Hospital of Fujian Medical University Department of Nuclear Medicine Fuzhou China Department of Nuclear Medicine, The First Affiliated Hospital of Fujian Medical University, Fuzhou, China; 4 The First Affiliated Hospital of Fujian Medical University Fujian Key Laboratory of Precision Medicine for Cancer Fuzhou China Fujian Key Laboratory of Precision Medicine for Cancer, The First Affiliated Hospital of Fujian Medical University, Fuzhou, China

**Keywords:** Kidney Neoplasms, Carcinoma, Renal Cell, gallium 68 PSMA-11 [Supplementary Concept]

## Abstract

**Objectives:**

Accurate preoperative prediction of adverse pathology is crucial for treatment planning of renal cell carcinoma (RCC). Previous studies have emphasized the potential of prostate-specific membrane antigen positron emission tomography / computed tomography (PSMA PET/CT) in differentiating between benign and malignant localized renal tumors. However, there is a scarcity of case reports elucidating the identification of aggressive pathological features using PET/CT. Our study was designed to prospectively compare the diagnostic value of enhanced CT, ^68^Ga-PSMA-11 and ^18^F-fluorodeoxyglucose (^18^F-FDG) PET/CT in clear-cell renal cell carcinoma (ccRCC) with necrosis or sarcomatoid or rhabdoid differentiation.

**Materials and Methods:**

A prospective case series of patients with a newly diagnosed renal mass who underwent enhanced CT, ^68^Ga-PSMA-11 and ^18^F-FDG PET/CT within 30 days prior to nephrectomy was included. Complete preoperative and postoperative clinicopathological data were recorded. Patients who received neoadjuvant targeted therapy, declined enhanced CT or PET/CT scanning, refused surgical treatment or had non-ccRCC pathological indications were excluded. Radiological parameters were compared within subgroups of pathological characteristics. Bonferroni corrections were used to adjust for multiple testing and statistical significance was set at a p-value less than 0.017.

**Results:**

Seventy-two patients were available for the final analysis. Enhanced CT demonstrated poor performance in identifying necrosis, sarcomatoid or rhabdoid differentiation and adverse pathology (all P > 0.05). The maximum standardized uptake value (SUVmax) of ^68^Ga-PSMA-11 PET/CT was more effective than ^18^F-FDG PET/CT in identifying tumor necrosis and adverse pathology, with an area under the curve (AUC) of 0.85 (cutoff value=25.26, p<0.001; Delong test z=2.709, p=0.007) for tumor necrosis and AUC of 0.90 (cutoff value=25.26, p<0.001; Delong test z=3.433, p<0.001) for adverse pathology. However, no significant statistical difference was found between ^68^Ga-PSMA-11 and ^18^F-FDG PET/CT in predicting sarcomatoid or rhabdoid feature (AUC of 0.91 vs.0.75, Delong test z=1.998, p=0.046). Subgroup analyses based on age, sex, tumor location, maximal diameter, stage and WHO/ISUP grade demonstrated that ^68^Ga-PSMA-11 PET/CT SUVmax had a significant predictive value for adverse pathology. Enhanced CT value and SUVmax demonstrated strong reliability [intraclass correlation coefficient (ICC) > 0.80], indicating a robust correlation.

**Conclusions:**

^68^Ga-PSMA-11 PET/CT demonstrates distinct advantages in identifying aggressive pathological features of primary ccRCC when compared to enhanced CT and ^18^F-FDG PET/CT. Further research and assessment are warranted to fully establish the clinical utility of ^68^Ga-PSMA-11 PET/CT in ccRCC.

## INTRODUCTION

In recent years, the incidence rate of renal cell carcinoma (RCC) has been increasing, ranking as the 9th most frequently diagnosed cancer in women and the 6th in men (1). Clear-cell RCC (ccRCC) is the main histologic subtype, accounting for 78.6% of all RCC tumors (2). Tumor necrosis (3), sarcomatoid (4) or rhabdoid differentiation (5) are well recognized as unfavorable outcomes, which are frequently observed in ccRCC. Immune checkpoint therapy (ICT) has exhibited encouraging outcomes in these subtypes (6), emphasizing the significance of precise preoperative prediction of aggressive pathological features to optimize treatment planning and enhance long-term survival. Although percutaneous biopsy can accurately diagnose pathological types, it carries risks of invasiveness and potential non-diagnostic results. Moreover, the intratumoral heterogeneity in ccRCC increases the likelihood of false negatives for the detection of adverse pathology due to spatial sampling bias (7).

The detection efficiency of ccRCC has been greatly enhanced owing to the extensive application of various imaging modalities (8). However, conventional imaging studies, such as enhanced computed tomography (CT), have only moderate accuracy in predicting histology (9). Molecular imaging with positron emission tomography (PET) potentially offers a more sensitive and specific alternative (10). ^18^F-fluoro-2-deoxy-2-d-glucose (FDG) PET/CT, a critical molecular imaging modality, has been used to evaluate RCC since the 1990s (11). However, its usefulness in characterizing renal masses has shown heterogeneity and limited benefits over CT (12), leading to its exclusion from routine practice guidelines for initial RCC diagnosis.

Prostate-specific membrane antigen (PSMA) PET/CT has been validated as a method to determine the benignity/malignancy of localized renal tumors and the WHO/ISUP grade of ccRCC (13, 14). Subsequently, Spatz et.al confirmed the prognostic value of PSMA in ccRCC at the protein level. However, case reports elucidating the identification of aggressive pathological features using PET/CT remain scarce (15). In this prospective study, we aim to compare the diagnostic value of enhanced CT, ^68^Ga-PSMA-11 and ^18^F-FDG PET/CT in ccRCC patients with adverse pathology and investigate the associations between radiological and pathological characteristics in these patients.

## MATERIALS AND METHODS

### Study design and patients

This prospective case series study was approved by the Ethics Committee of the Medical University. The study protocol was registered in the Chinese Clinical Trial Registry (www.chictr.org.cn; registration number: ChiCTR2100044927). Written informed consents were obtained from all included patients. Pathological tumor characteristics and radiographic tumor features were collected. The inclusion criteria were as follows: (1) enhanced CT, ^68^Ga-PSMA-11 and ^18^F-FDG PET/CT performed within 30 days before surgery; (2) complete preoperative and postoperative clinicopathological data; (3) underwent partial, radical, or cytoreductive nephrectomy. The exclusion criteria were as follows: (1) radiotherapy, targeted therapy, immunotherapy, or other neoadjuvant treatment before operation; (2) refusal of enhanced CT or PET/CT imaging; (3) refusal of surgical treatment; (4) pathological results suggesting urothelial carcinoma, benign tumor or other renal malignancies.

### Enhanced CT examination

All CT imaging was performed using Aquilion ONE 320-detector row helical scanners (Canon Medical Systems™). Patients were scanned in a supine position and asked to hold their breath during the imaging procedure. Enhanced CT examination was performed by injecting iohexol through the elbow vein via a high-pressure syringe at a flow rate of 3.0 mL/s. Corticomedullary phase (CMP) was acquired 30s after contrast injection, nephrographic phase (NP) at 80s and excretory phase (EP) at ^18^0s after injection.

### ^68^Ga-PSMA-11 PET/CT exam

All patients received an intravenous injection of ^68^Ga-PSMA-11 (^68^Ga-HBED-CC-11-PSMA) tracer. PET/CT imaging was performed using a PET/CT scanner (Biograph mCT64, Siemens Healthcare™). Approximately 40-60 minutes after injecting ^68^Ga-PSMA-11 (1.85 MBq/kg), a non-contrast-agent CT scan was acquired from the top of the skull to the level of the middle leg (120 keV, 80 mAs, slice thickness 3.0 mm). Static emission scanned PET images were acquired in three dimensions (matrix 200×200) from the apex to the proximal leg, with corrections applied for dead time, scattering and attenuation. A total of 6-8 bed positions (each for two minutes) were acquired. Images were reconstructed iteratively (2 iterations and 21 subsets) using an ordered subset expectation maximization (OSEM) algorithm with CT-based attenuation correction. PET/CT images were co-registered and displayed by dedicated software (TrueD software, Siemens™).

### ^18^F-FDG PET/CT examination

Patients were required to fast for at least 6 hours prior to examination. ^18^F-FDG PET/CT imaging was performed using a PET/CT scanner (Biograph mCT64, Siemens Healthcare™). Approximately 40-60 minutes after intravenous injection of ^18^F-FDG at a dose of 3.7 MBq/kg, low-dose CT was acquired for attenuation correction and accurate anatomical positioning (tube voltage, 120 kV; tube current, automatic mA). Patients were instructed to maintain shallow breathing during the scan and three-dimensional imaging was obtained from the top of the skull to the middle leg level, with a duration of 2 minutes per bed position. The PET images were reconstructed iteratively with attenuation correction.

### PET/CT imaging analyses

All PET/CT images were independently reviewed and analyzed by two nuclear physicians, including a double-trained board-certified physician. Any discrepancies between the readers were resolved through consensus reading. The following features were assessed: (a) lesion count; (b) lesion localization; (c) presence of focal radiotracer uptake. PET/CT fusion images were constructed, and regions of interest (ROIs) were drawn around the contours of the primary tumors on continuous axial fusion images. To minimize the influence of normal kidney tissues, ROIs were drawn in distal parts of the tumor. Additionally, a normal background ROI with a diameter of 3 cm was drawn in non-tumor normal liver tissue of the right lobe of the liver. The maximum standardized uptake value (SUVmax), mean SUV (SUVmean) and peak SUV (SUVpeak) of each primary tumor and normal background liver were measured, and the tumor-to-liver SUV ratio (TLR) was calculated.

### CT scanning evaluation

The CT images were independently scrutinized by two experienced radiologists who were blinded to the clinicopathological data. Any disagreements were resolved through consensus reading. To standardize the evaluation of tumor enhancement, ROIs were selected to observe the changes in CT values during different scan phases. Since the tumor enhancement could be better visualized on the corticomedullary phase (CMP) images and the various heterogenous components of the tumor could be observed at this stage, all enhanced CT values in this study were measured based on the CMP images.

### Histopathology analysis

After nephrectomy, renal tumor specimens were fixed in formalin, embedded in paraffin, step-sectioned at 3–4 μm intervals, and mounted on treated glass slides. At least 10 blocks were selected for each case. For large tumors, at least one block was selected every one centimeter of the tumor. The percentage of necrosis, sarcomatoid or rhabdoid differentiation was recorded. Hematoxylin and eosin (H&E) staining was routinely performed and assessed microscopically. The pathological features of the tumor were evaluated. T1 and T2 ccRCC were defined as localized ccRCC, while T3 and T4 ccRCC were defined as locally advanced ccRCC (16). The presence of tumor necrosis (3) and sarcomatoid (4) and rhabdoid (5) features was defined as adverse pathology.

### Statistical Analyses

SPSS 26.0 (IBM Corp, Armonk, NY, USA) and MedCalc version 15.2 (Ostend, Belgium) were used for statistical analyses. Normally distributed data were expressed as mean ± standard deviation (SD) and analyzed using Student's t-test, while non-normally distributed data were expressed as median and interquartile range (IQR) and analyzed using the Mann-Whitney U test. Receiver operating characteristics (ROC) curves were constructed to identify optimal cutoff values and evaluate sensitivity, specificity, positive predictive value (PPV), and negative predictive value (NPV) based on the maximal Youden index. The area under the ROC curve (AUC) and its 95% confidence intervals (CIs) were calculated. DeLong' test was used to compare the AUCs of different variables (17). Interobserver agreement for PET/CT and CT image evaluations were assessed using intraclass correlation coefficients (ICCs), categorized as poor (less than 0.20), fair (0.21-0.40), moderate (0.41-0.60), substantial (0.61-0.80) or almost perfect agreement (0.81-1.00) (18). Bonferroni corrections were applied to adjust for multiple testing and a two-sided p-value <0.017 was considered statistically significant.

## RESULTS

### Demographics and Clinical Data of ccRCC

**Figure 1:**
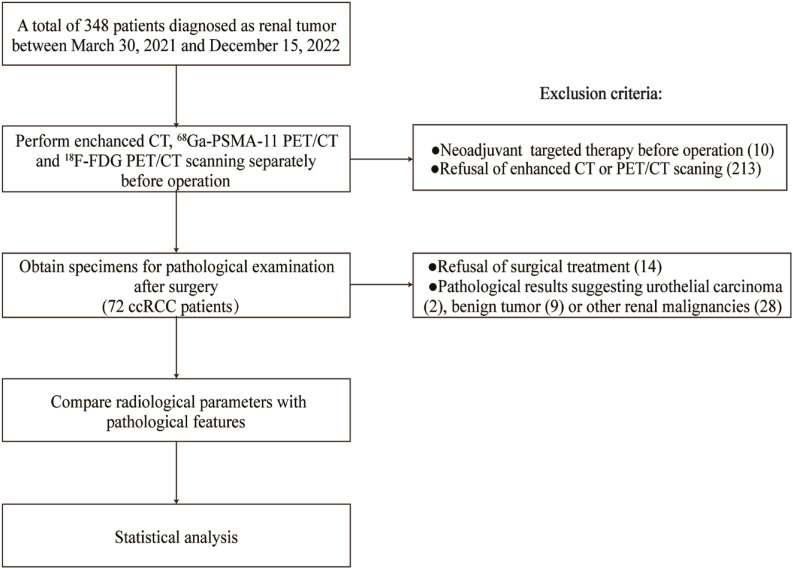
Flow chart of patient recruitment and study procedures.

A total of 348 patients were initially included in the database between March 30th, 2021, and December 15th, 2022. Ten patients were excluded due to neoadjuvant targeted therapy, two hundred and thirteen were excluded because they refused enhanced CT or PET/CT scanning, four were excluded because of the refusal of surgical treatment and others because their pathological results were non-ccRCC (two cases of urothelial carcinoma, nine of benign tumor and twenty-eight of other renal malignancies (Figure-1). Seventy-two patients were available for the final analysis, and their demographics and clinical data are shown in Table 1. The median age was 57 years (IQR 51-66). Tumor necrosis was present in 13 patients (18.1%), sarcomatoid or rhabdoid feature in 6 (8.3%), adverse pathology in 17 (23.6%) and vascular cancer embolus in 8 (11.1%). Among the malignant lesions, 39 (54.2%) were categorized as localized ccRCC and 33 (45.8%) as locally advanced ccRCC. The distribution of WHO/ISUP grades was as follows: grade 1, 11 (15.3%); grade 2, 33 (45.8%); grade 3, 15 (20.8%); and grade 4, 13 (18.1%).

**Table 1:** Demographics and Clinical Characteristics of the 72 Patients with ccRCC

	Parameter	Value
**Age (y)**		57(51-66)
**Tumor maximal diameter (cm)**		6.7(4.1-10.2)
**Sex, (%)**		
	Male	38(52.7)
	Female	34(47.3)
**Tumor location, (%)**		
	Left	33(45.8)
	Right	39(54.2)
**Vascular cancer embolus, (%)**		
	Positive	8(11.1)
	Negative	64(88.9)
**Tumor necrosis, (%)**		
	Positive	13(18.1)
	Negative	59(81.9)
**Sarcomatoid or rhabdoid feature, (%)**		
	Positive	6(8.3)
	Negative	66(91.7)
**Adverse pathology, (%)** *		
	Positive	17(23.6)
	Negative	55(76.4)
**pT stage, (%)**		
	T1	26(36.1)
	T2	13(18.1)
	T3	22(30.6)
	T4	11(15.2)
**WHO/ISUP grade, (%)**		
	1	11(15.3)
	2	33(45.8)
	3	15(20.8)
	4	13(18.1)

* Adverse pathology: tumor presenting with necrosis or a sarcomatoid or rhabdoid feature. Two of the tumor has both tumor necrosis and sarcomatoid feature.

ccRCC = clear-cell renal cell carcinoma; WHO/ISUP = World Health Organization / International Society of Urological Pathology

Data are median (interquartile range) for age and tumor maximal diameter.

### Relationships of Enhanced CT and PET/CT Imaging Features for Identification of Pathological Characteristics in ccRCC

**Figure 2:**
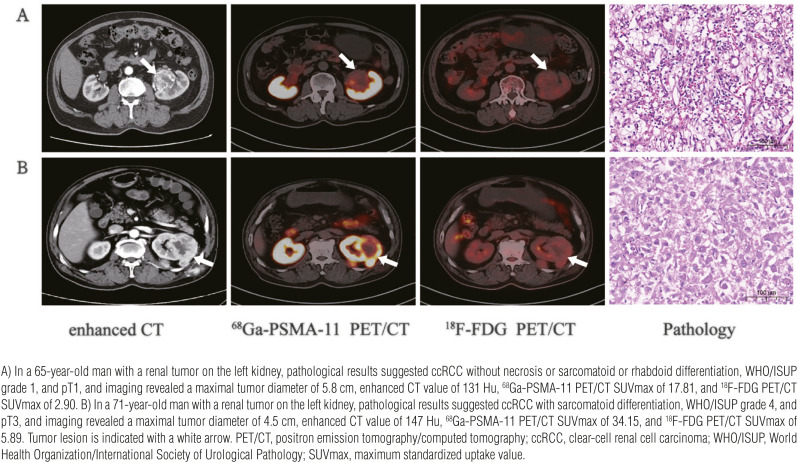
Representative images depicting radio-pathological matched cases, exemplifying variations in radiotracer uptake in ccRCC with different degrees of differentiation.

To explore the diagnostic performance of enhanced CT and PET/CT imaging for discriminating pathological characteristics of ccRCC, we derived radiological parameters including CT value from enhanced CT scanning and SUV from PET/CT images. Representative radio-pathological matching cases are shown in Figure-2. Several radiological parameters were compared by subgroups of pathological characteristics (Table-2 and Supplementary Table-1). The ICCs between the two inter-observers were 0.935 [95% CI (0.959-0.975)] for enhanced CT value, 0.972 [95% CI (0.955-0.983)] for ^68^Ga-PSMA-11 PET/CT and 0.911 [95% CI (0.862-0.943)] for 18F-FDG PET/CT for all cases. In all three subgroups of pathological characteristics, the ICCs were 0.8 or greater, indicating an almost perfect correlation (Table-2).

**Table 2:** Relevance of Enhanced CT, ^68^Ga-PSMA-11 PET/CT and ^18^F-FDG PET/CT Parameters for Identification of Pathological Characteristics in ccRCC.

Pathological features		Radiological parameters								
		Enhanced CT			^68^Ga-PSMA-11 PET/CT			^18^F-FDG PET/CT		
		CT value	ICC^a^	P^b^ value	SUV_max_	ICC^a^	P^c^ value	SUV_max_	ICC^a^	P^c^ value
**Tumor necrosis**				0.221			<0.001			0.119
	Negative	128.97± 31.86	0.936 [0.895-0.961]		17.39 (7.58-25.20)	0.969 [0.949-0.982]		3.97 (2.98-6.40)	0.906 [0.848-0.943]	
	Positive	141.08±32.87	0.929 [0.787-0.961]		30.51 (26.18-32.54)	0.898 [0.679-0.968]		5.92 (3.32-6.81)	0.922 [0.767-0.975]	
**Sarcomatoid or rhabdoid feature**				0.262			<0.001			0.038
	Negative	129.86 32.66	0.937 [0.899-0.961]		18.76 (8.91-26.93)	0.971 [0.952-0.982]		4.02 (3.03-6.79)	0.907 [0.852-0.942]	
	Positive	145.33± 23.48	0.895 [0.477-0.984]		33.93 (32.54-35.33)	0.837 [0.195-0.976]		6.79 (5.45-8.04)	0.914 [0.537-0.987]	
**Adverse pathology** *				0.179			<0.001			0.049
	Negative	128.31±32.63	0.937 [0.894-0.962]		14.94 (7.36-23.32)	0.967 [0.944-0.981]		3.69 (2.94-6.40)	0.915 [0.859-0.949]	
	Positive	140.35± 29.63	0.927 [0.811-0.973]		31.55 (27.87-34.39)	0.869 [0.683-0.950]		5.59 (4.52-6.81)	0.900 [0.745-0.963]	

* Adverse pathology is defined as tumor presenting with necrosis or a sarcomatoid or rhabdoid feature

a ICC: Data in the brackets are 95% confidence intervals

b P values were calculated with Student's t test

c P values were calculated with Mann-Whitney U test

ccRCC = clear-cell renal cell carcinoma; CT = computed tomography; ICC = Intraclass correlation coefficient; SUV_max_ = maximum standardized uptake value

Data are mean ± standard deviation for CT value, median (interquartile range) for SUV_max_

**Figure 3:**
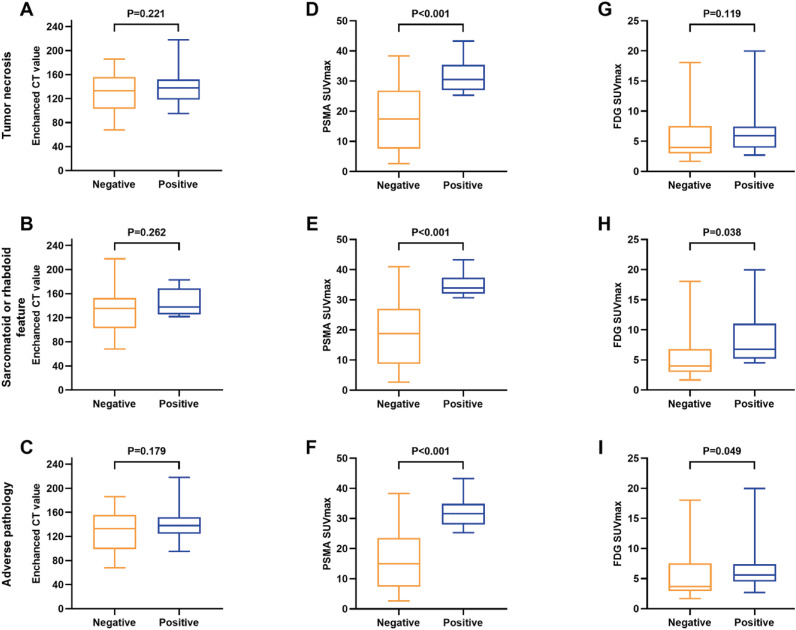
Box plot illustrating the distribution of enhanced CT value (A-C), ^68^Ga-PSMA-11 (D-F) and ^18^F-FDG PET/CT SUV_max_ (G-I) according to the pathological features of ccRCC. The pathological characteristics include tumor necrosis (A, D, G), sarcomatoid or rhabdoid differentiation (B, E, H) and adverse pathology (C, F, I). Adverse pathology is defined as a tumor presenting with necrosis or a sarcomatoid or rhabdoid feature; SUV_max_, maximum standardized uptake value; CT, computed tomography; ccRCC, clear-cell renal cell carcinoma.

As can be seen in Table-2, Figure-2 and Figura-3, enhanced CT values demonstrated poor performance in identifying tumor necrosis, sarcomatoid or rhabdoid feature and adverse pathology (all P>0.05) (Figure-2, Figures 3A, 3B and 3C). In contrast, ^68^Ga-PSMA-11 PET/CT SUVmax significantly differed by subgroups of tumor necrosis, sarcomatoid or rhabdoid feature and adverse pathology (all P<0.01) (Figure-2, Figures 3D, 3E and 3F). Among the four SUV parameters, SUVmax demonstrated more significant differences in most pathological characteristics compared to SUVmean, SUVpeak and TLR (Supplementary Table-1). As for ^18^F-FDG PET/CT, SUVmax did not significantly differed by subgroups of tumor necrosis (P=0.119), sarcomatoid or rhabdoid feature (P=0.038) and adverse pathology (P=0.049) (Table-2) (Figure-2, Figures 3G, 3H and 3I).

### Effectiveness of Radiological Parameters for Identifying Adverse Pathology of ccRCC

**Figure 4:**
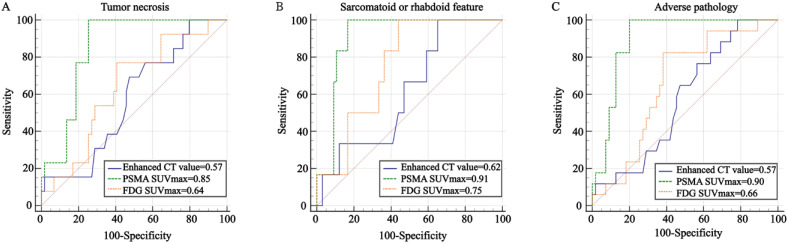
ROC curves comparing the effectiveness of radiological parameters in predicting the pathological characteristics of ccRCC. Radiological parameters: enhanced CT value, ^68^Ga-PSMA-11 and ^18^F-FDG PET/CT SUVmax; pathological characteristics: tumor necrosis, sarcomatoid or rhabdoid differentiation and adverse pathology. Adverse pathology is defined as a tumor presenting with necrosis or a sarcomatoid or rhabdoid feature; ROC, Receiver operating characteristics; AUC, area under the curve; SUV_max_, maximum standardized uptake value; CT, computed tomography; ccRCC, clear-cell renal cell carcinoma.

ROC analysis was used to evaluate the effectiveness of radiological parameters in identifying aggressive pathological features of ccRCC, including tumor necrosis (Figure-4A), sarcomatoid or rhabdoid feature (Figure-4B) and adverse pathology (Figure-4C). Enhanced CT value showed poor performance in identifying necrosis, sarcomatoid or rhabdoid feature and adverse pathology, with AUC values of 0.57, 0.62, and 0.57, respectively (all P > 0.05) (Table-3 and Figure-4). ^68^Ga-PSMA-11 PET/CT SUVmax performed better than ^18^F-FDG PET/CT SUVmax in identifying tumor necrosis with a PPV of 46%, NPV of 100% and the AUC values of 0.85 (95% CI, 0.76-0.94, P < 0.001; Delong test z=2.709, p<0.01) (Figure-4A). When the SUVmax cutoff value from the ROC curve for ccRCC with tumor necrosis was stratified by 25.26, the corresponding sensitivity and specificity were 100% and 75%, respectively (Table-3). When used to identify adverse pathology, the SUVmax of ^68^Ga-PSMA-11 PET/CT performed better than ^18^F-FDG PET/CT, with a PPV of 61% vs. 40%, NPV of 100% vs. 92% and the AUC value of 0.90 (95% CI, 0.83-0.97) vs. 0.66(95% CI, 0.52-0.79) (Delong test, z=3.433, p<0.001) (Figure-4C). With the specific cutoff value of 25.26, ^68^Ga-PSMA-11 PET/CT SUVmax showed sensitivity 100% and specificity 80% for adverse pathology (positive vs. negative). Moreover, there was no significant statistical difference between ^68^Ga-PSMA-11 and ^18^F-FDG PET/CT in predicting sarcomatoid or rhabdoid feature, with a specificity of 83% vs. 56%, PPV of 35% vs.17% and the AUC value of 0.91 (95% CI, 0.84-0.98) vs. 0.75(95% CI, 0.61-0.90) (Delong test, z=1.998, p=0.046) (Figure-4B). Subgroup analyses based on age, sex, tumor location, tumor maximal diameter, tumor stage and WHO/ISUP grade consistently showed that ^68^Ga-PSMA-11 PET/CT SUVmax had a significant predictive value for adverse pathology (Supplementary Table-2).

**Table 3:** Effectiveness of Enhanced CT, ^68^Ga-PSMA-11 and ^18^F-FDG PET/CT Parameters for Identifying the Pathological Features of ccRCC Patients by ROC Curve Analysis.

Pathological features (positive vs. negative)	Radiological parameters	AUC (95%CI)	Cutoff value	Sensitivity	Specificity	PPV	NPV	P value
Tumor necrosis	CT value	0.57(0.42-0.73)	94.00	1.00	0.20	0.22	1.00	0.221
	SUV_max_ (^68^Ga-PSMA-11 PET/CT)	0.85(0.76-0.94)	25.26	1.00	0.75	0.46	1.00	<0.001
	SUV_max_ (^18^F-FDG PET/CT)	0.64(0.48-0.79)	4.54	0.77	0.59	0.29	0.92	0.119
Sarcomatoid or rhabdoid feature	CT value	0.62(0.42-0.82)	118.00	1.00	0.35	0.12	1.00	0.262
	SUV_max_(^68^Ga-PSMA-11 PET/CT)^a^	0.91(0.84-0.98)	30.58	1.00	0.83	0.35	1.00	<0.001
	SUV_max_(^18^F-FDG PET/CT)^a^	0.75(0.61-0.90)	4.52	1.00	0.56	0.17	1.00	0.038
Adverse pathology*	CT value	0.57(0.43-0.72)	94.00	1.00	0.22	0.28	1.00	0.179
	SUV_max_(^68^Ga-PSMA-11 PET/CT)^b^	0.90(0.83-0.97)	25.26	1.00	0.80	0.61	1.00	<0.001
	SUV_max_(^18^F-FDG PET/CT)^b^	0.66(0.52-0.79)	4.52	0.82	0.62	0.40	0.92	0.049

a SUV_max_(^68^Ga-PSMA-11 PET/CT) vs. SUV_max_(^18^F-FDG PET/CT) in distinguishing sarcomatoid or rhabdoid feature (Delong test, z=1.998, p=0.046)

b SUV_max_(^68^Ga-PSMA-11 PET/CT) vs. SUV_max_(^18^F-FDG PET/CT) in distinguishing adverse pathology (Delong test, z=3.433, p<0.001)

* Adverse pathology is defined as tumor presenting with necrosis or a sarcomatoid or rhabdoid feature

Units: CT value, Hu ccRCC = clear-cell renal cell carcinoma; CT = computed tomography; SUV_max_ = maximum standardized uptake value; AUC = area under the ROC curve; CI = confidence interval; PPV = positive predictive value; NPV = negative predictive value

## DISCUSSION

As an immunologically and histologically heterogeneous tumor, ccRCC exhibits diverse prognoses and responses to systemic therapy. In previous studies, necrosis (3) and differentiation (including sarcomatoid (4) or rhabdoid (5) feature) within the tumor specimen have been reported as adverse prognostic factors in patients with RCC, independently associated with worse survival. The utility and timing of cytoreductive nephrectomy were questioned by results from the CARMENA and SURTIME trials, respectively (19, 20). Early stage localized small RCC responds well to partial nephrectomy, but for those with aggressive pathological features, radical nephrectomy or postoperative combination immunotherapy is often required. Therefore, exploring approaches to the accurate preoperative prediction of these aggressive pathological features will be helpful in therapeutic decision-making and clinical management. Deutsch et al. (21) recently reported a mutiparametric method, including tumor necrosis, for predicting outcomes to anti-PD-1 therapy in ccRCC. Currently, there is great debate with inconsistent data demonstrating the efficacy of adjuvant immunotherapy in resected high-risk ccRCC. However, it is clear that immunotherapy treatments are more effective in sarcomatoid disease (reasonable extrapolation from data in the metastatic setting) - hence the critical need to have predictive biomarkers in this setting (6).

Currently, contrast-agent-enhanced CT and MRI are the most commonly used diagnostic imaging methods for ccRCC. However, some studies have shown that these two imaging diagnostic methods have limitations in the early diagnosis of ccRCC (22-24). The retrospective study of Oh et al. (22) found no significant correlation between CT findings of ccRCC and Fuhrman grade. Zhu et al. (23) found enhanced CT had no statistically significant difference in identifying sarcomatoid ccRCC, with a P-value of 0.09. For MRI, Tsili et al. (24) found that diffusion-weighted imaging (DWI), which can be used to predict RCC histologic grade, had limitations in the effectiveness of qualitative analysis of renal parenchymal masses due to significant overlapping of apparent diffusion coefficient (ADC) values among different histologic subtypes, further limiting its use in clinical activity in the early diagnosis of ccRCC. In this context, PET/CT imaging, with its higher sensitivity and specificity, becomes an appealing option.

Namura et al. (25) considered that ^18^F-FDG-PET/CT had potency as an "imaging biomarker" for providing valuable information for clinical decision-making. In addition, as products of its folate hydrolase activity are associated with angiogenesis, the highly vascularized nature of ccRCC makes it a potential PSMA-avid tumor (26). There is also preliminary evidence suggesting that PSMA PET/CT may be valuable in staging, restaging and response assessment in ccRCC (13, 14). Unfortunately, the molecular imaging phenotype of ccRCC with aggressive pathological features is less well established, with only limited case reports and retrospective studies describing intense ^18^F-FDG uptake within rapidly progressive sarcomatoid sites on ^18^F-FDG PET/CT. Liang et al. (27) reported that sarcomatoid ccRCC with high SUVmax showed more aggressive biological behavior through case reports. Zhu et al. (23) found that it was helpful to indicate the sarcomatoid differentiation of ccRCC when FDG PET/CT SUVmax, SUVmean and SUVpeak cutoff values of 5.4, 4.2 and 5.0 respectively, which were consistent with our statistical analysis results. In the current study, ^18^F-FDG PET/CT exhibited limited ability to distinguish tumor necrosis in ccRCC, whereas ^68^Ga-PSMA-11 PET/CT demonstrated superior discriminatory capabilities.

To our knowledge, this is the first prospective study comparing the diagnostic value of enhanced CT, ^68^Ga-PSMA-11 and ^18^F-FDG PET/CT parameters in ccRCC with necrosis or sarcomatoid or rhabdoid differentiation. Our results indicate that enhanced CT had limited performance in identifying necrosis, sarcomatoid or rhabdoid feature and adverse pathology in ccRCC. Moreover, ^68^Ga-PSMA-11 PET/CT SUVmax demonstrated a sensitivity of 100% and specificity of 75% for discriminating tumor necrosis, and a sensitivity of 100% and specificity of 80% for adverse pathology, suggesting that ^68^Ga-PSMA-11 PET/CT performs better than enhanced CT and ^18^F-FDG PET/CT in identifying tumor necrosis and adverse pathology. These findings align with previous case reports and retrospective cohort studies (15, 28), emphasizing the advantage of receptor ligand-targeted molecular imaging with its higher specificity compared to metabolic uptake represented by FDG.

Although there are only a few studies of PSMA PET/CT in renal tumors, an increasing number of scholars are recognizing its growing role (29). In a retrospective study involving 36 ccRCC patients, Gao et al. (14) concluded that ^68^Ga-PSMA-11 PET/CT SUVmax could effectively identify adverse pathology with an AUC of 0.92 (cutoff value=^18^.5, P<0.001). Nadebaum et al. (15) demonstrated intense ^68^Ga-PSMA uptake within the site of renal tumor with sarcomatoid feature through quantitative analysis of clinical images (SUVmax=16.1). These research findings are consistent with our preliminary conclusions. Additionally, we observed that ^68^Ga-PSMA-11 PET/CT SUVmax values were also increased in ccRCC with necrosis (30.51 vs.17.39, P<0.001). Consequently, in clinical practice, patients who undergo preoperative ^68^Ga-PSMA-11 PET/CT scanning and exhibit intense SUVmax values should be highly alert to contain aggressive pathological features. Such patients ought to receive more comprehensive and radical treatment, along with increased caution when dealing with advanced diseases.

There are several limitations in our study. First, the sample size was relatively small. Further research with a larger sample size and involving multiple centers is necessary to validate our findings. Second, imaging PSMA in the kidney remains challenging due to its high uptake in that organ. Third, the drawing of ROIs was subjective as it depended on the observer's evaluation and could introduce variability. Fourth, a considerable number of patients were excluded because of the refusal of enhanced CT or PET/CT scanning, which could potentially introduce selection bias and affect the generalizability of the results. Finally, the lack of a reference standard diagnostic method might impact the diagnostic value of ^68^Ga-PSMA-11 and ^18^F-FDG PET/CT.

## CONCLUSIONS

^68^Ga-PSMA-11 PET/CT offers distinct advantages over enhanced CT and ^18^F-FDG PET/CT in identifying adverse pathology in primary ccRCC. This non-invasive imaging modality shows potential for aiding in early decision-making, evaluating treatment efficacy and predicting the risk of adverse outcomes in ccRCC patients. The findings support the potential clinical application of ^68^Ga-PSMA-11 PET/CT in ccRCC patients' management. Further research with larger patient cohorts is warranted to validate these findings and explore its full clinical utility.

## Data Availability

The datasets used and/or analyzed during the current study are available from the corresponding author on reasonable request.

## APPENDIX:

**Supplementary Table 1:** Association between PET parameters and pathological characteristics of ccRCC.

		^68^Ga-PSMA-11 PET/CT								^18^F-FDG PET/CT							
		SUV_max_	P^a^ value	SUV_mean_	P^a^ value	SUV_peak_	P^a^ value	TLR	P^a^ value	SUV_max_	P^a^ value	SUV_mean_	P^a^ value	SUV_peak_	P^a^ value	TLR	P^a^ value
**Tumor necrosis**			<0.001		0.016		0.026		0.017		0.119		0.057		0.107		0.241
	Negative	17.39		9.01		10.72		2.17		3.97		2.51		2.95		1.26	
		7.58-25.20)		(4.04-13.68)		(5.39-18.44)		(1.32-3.93)		(2.98-6.40)		(1.82-3.48)		(2.33-4.95)		(1.04-1.96)	
	Positive	30.51		15.39		20.79(16.91-23.47)		4.15		5.92		3.79		4.15		1.96	
		(26.18-32.54)		(12.58-19.25)				(3.29-4.81)		(3.32-6.81)		(2.57-4.49)		(2.88-5.61)		(1.16-2.06)	
**Sarcomatoid or rhabdoid feature**			<0.001		0.001		0.003		0.001		0.038		0.148		0.126		0.089
	Negative	18.76		10.23		13.91		2.64		4.02		2.69		3.44		1.26	
		(8.91-26.93)		(5.06-13.21)		(6.85-19.35)		(1.50-4.03)		(3.03-6.79)		(1.83-4.05)		(2.34-5.38)		(1.04-2.06)	
	Positive	33.93		15.67		20.80(19.74-22.74)		4.82		6.79		3.55		4.71		1.82	
		(32.54-35.33)		(15.39-18.53)				(4.39-4.87)		(5.45-8.04)		(2.56-6.31)		(3.57-7.11)		(1.61-2.50)	
**Adverse pathology** *			<0.001		0.001		0.001		0.001		0.049		0.055		0.079		0.15
	Negative	14.94		8.39		9.89		2.1		3.69		2.35		2.76		1.23	
		(7.36-23.32)		(4.02-12.65)		(5.22-23.52)		(1.20-3.39)		(2.94-6.40)		(1.77-3.48)		(2.32-4.95)		(1.04-2.17)	
	Positive	31.55		15.48		20.79		4.3		5.59		2.99		4.44		1.68	
		(27.87-34.39)		(10.21-15.86)		(18.02-22.74)		(3.51-4.82)		(4.52-6.81)		(2.56-4.49)		(2.95-5.61)		(1.33-2.01)	

* Adverse pathology is defined as tumor presenting with necrosis or a sarcomatoid or rhabdoid feature

a P values were calculated with Mann-Whitney U test

ccRCC = clear-cell renal cell carcinoma; SUV_max_ = maximal standard uptake value; SUV_mean_ = mean standard uptake value; SUV_peak_ = peak standard uptake value; TLR = tumor-to-liver standard uptake value ratio

Data are median (interquartile range) for SUV_max_, SUV_mean_, SUV_peak_ and TLR

**Supplementary Table 2:** Subgroup analyses of ^68^Ga-PSMA-11 PET/CT SUVmax in patients with or without adverse pathology.

Factors	Variable	^68^Ga-PSMA-11 PET/CT SUVmax		P^a^ value
		Without adverse pathology*	With adverse pathology	
Age (y)	≤ 57	14.57(8.25-26.89)	32.03(28.19-34.39)	<0.001
	> 57	14.39(6.43-19.24)	29.98(27.87-32.54)	<0.001
Sex	Male	13.83(6.26-19.12)	31.55(28.19-33.47)	<0.001
	Female	17.01(8.91-26.81)	31.00(26.18-34.39)	0.008
Tumor location	Left	13.99(6.43-19.55)	32.54(29.45-35.33)	<0.001
	Right	14.94(8.25-23.32)	29.19(25.78-32.03)	0.002
Tumor maximal diameter (cm)	≤ 6.7	11.49(6.71-14.99)	30.51(26.18-32.03)	<0.001
	> 6.7	20.44(7.58-26.93)	33.47(29.45-35.33)	0.001
Tumor stage	T1-T2	10.39(6.26-17.39)	26.18(25.31-28.19)	<0.001
	T3-T4	23.52(14.94-34.97)	33.00(30.64-35.33)	0.003
WHO/ISUP grade	01-Feb	10.39(6.26-14.99)	27.03(25.31-29.45)	0.001
	03-Apr	26.93(23.52-32.98)	33.47(31.55-35.33)	0.015

* Adverse pathology is defined as tumor presenting with necrosis or a sarcomatoid or rhabdoid feature

a P values were calculated with Mann-Whitney U test

SUV_max_ = maximum standardized uptake value; WHO/ISUP = World Health Organization / International Society of Urological Pathology

Data are median (interquartile range) for SUV_max_
